# Root colonization dynamics of alginate encapsulated rhizobacteria: implications for *Arabidopsis thaliana* root growth and durum wheat performance

**DOI:** 10.3934/microbiol.2025006

**Published:** 2025-02-05

**Authors:** Amel Balla, Allaoua Silini, Hafsa Cherif-Silini, Francesca Mapelli, Sara Borin

**Affiliations:** 1 Laboratory of Applied Microbiology, Department of Microbiology, Faculty of Natural and Life Sciences, University Ferhat Abbas of Setif -1, 19000 Setif, Algeria; 2 Department of Food, Environmental and Nutritional Sciences (DeFENS), University of Milan, 20133 Milan, Italy

**Keywords:** bioencapsulation, sodium alginate, PGPB, *Arabidopsis thaliana*, durum wheat, root colonization

## Abstract

Bioencapsulation in alginate capsules offers an interesting opportunity for the efficient delivery of microbial inoculants for agricultural purposes. The present study evaluated the ionic gelation technique to prepare beads loaded with two plant growth-promoting bacteria (PGPB), *Bacillus thuringiensis* strain B25 and *Pantoea agglomerans* strain Pa in 1% alginate supplemented with 5mM proline as an osmoprotectant. Capsule morphology, survival rate, encapsulation efficiency, and viability during 24 months of storage as well as the stability of PGP activities were studied. Our results indicate that more than 99% of bacteria were effectively trapped in the alginate beads, which successfully released live bacteria after 60 days of storage at room temperature. A considerable survival of *B. thuringiensis* B25 throughout the storage period was detected, while the inoculated concentration of 8.72 × 10^9^ (±0.04 ×10^9^) CFU/mL was reduced to 99.9% for *P. agglomerans* Pa after 24 months of storage. Notably, a higher survival of individually encapsulated bacteria was observed compared to their co-inoculation. The colonization capacity of model plant *Arabidopsis thaliana* roots by free and encapsulated bacteria was detected by the triphenyltetrazolium chloride test. Moreover, both strains effectively colonized the rhizosphere, rhizoplane, and endosphere of durum wheat plants and exerted a remarkable improvement in plant growth, estimated as a significant increase in the quantities of total proteins, sugars, and chlorophyll pigments, besides roots and shoots length. This study demonstrated that alginate-encapsulated *B. thuringiensis* B25 and *P. agglomerans* Pa could be used as inoculants in agriculture, as their encapsulation ensures robust protection, maintenance of viability and PGP activity, and controlled bacterial biostimulant release into the rhizosphere.

## 1. Introduction

It is predicted that approximately 9.7 billion people will inhabit Earth by 2050 [1]; this demographic expansion, inextricably combined with massive industrialization and climate change, poses major threats to global food security [2]. In response to the growing food needs and desires of the population, the use of fertilizers and pesticides is an inevitable approach to ensure crop productivity and yield. Around 2.7 million tons of pesticides [3] and 190 million tons of synthetic chemical fertilizers [2] are used yearly worldwide. However, overexploitation of agricultural land and the increased and uncontrolled use of chemicals endanger the entire terrestrial ecosystem, causing problems to human and animal health [4] Furthermore, the contamination of soil, water, and food andthe degradation of soil fertility and biological balance of the entire ecosystem [5], as well as a reduction in soil nutrient content and a weakening of crops against various pathogens and pests [6], can be generated. To manage these threats, the scientific community is exploring solutions to protect the environment and minimize the use of synthetic chemicals in agricultural practices. Sustainable agriculture via microbial bioengineering has attracted significant attention in the last decade as a non-invasive and environmentally friendly alternative [7]. The application of PGPB (plant growth-promoting bacteria) as bioinoculants (biofertilizers, biostimulants, or biopesticides) is a promising approach aimed at ensuring healthy production and maintenance of soil quality and biodiversity [8]. PGPBs have immense potential to improve plant growth, abiotic stress tolerance, and pathogen suppression. Such potential is related to their ability to boost plant access to nutrients, for instance by recycling and solubilizing minerals [9] and to produce phytohormones and growth regulators [10]. PGPBs can also control diseases spread in the soil by induced systemic resistance (ISR) and by secreting various antimicrobial molecules [11].

A large number of bacteria are known for their ability to exert plant probiotic effects. *Pantoea agglomerans* is one of the most promising bacteria in agriculture, being known as a colonizer of various cereals, notably wheat [12] and maize [13]. *Pantoea agglomerans* has an arsenal of attributes that make it a robust PGPB such as phytohormone production [14], phosphate solubilization, ammonia production [15], secretion of various proteins and enzymes [16], and the stimulation of the plant immune system, as it has the capacity to fight against various fungal phytopathogens [17]. The bacterial species *Bacillus thuringiensis* is a marketed biopesticide known as a biocontrol agent effective against several pests [18]. This sporulating bacterium also has remarkable biofertilization and biostimulation properties: it produces phytohormones, ACC deaminase, and siderophores, solubilizes phosphate, and synthesizes volatile organic compounds [19].

Despite literature reports on the immense potential of PGPBs in improving plant growth and yield and mitigating the effects of biotic and abiotic stress, and despite the successful development of liquid bioformulations, the market share of biofertilizers represents only 5% of the global fertilizer market [20]. The application and commercialization of bioformulations encounter many challenges, such as conservation, viability, and contamination issues, cost, and sensitivity to environmental factors [21]. The encapsulation of bacterial cells or immobilization in biodegradable, biocompatible, non-toxic, and low-cost biopolymers such as alginate aims to protect bacteria introduced into the soil against biotic and abiotic stress in order to ensure the preservation of their activities. This strategy can also guarantee maximum viability and progressive cell release into the rhizosphere for efficient root colonization [22], according to the principle of precision agriculture. However, current literature offers limited information on the persistence and effectiveness of activities related to plant growth promotion after bacterial cell encapsulation. This process may face major challenges; hence, clarifications are necessary.

In this study, *Arabidopsis thaliana* and *Triticum durum* (durum wheat) were selected to evaluate the efficacy of encapsulated bacteria. *Arabidopsis thaliana*, as a universal model plant, offers a rapid and controlled experimental platform to study plant–microbe interactions [23], while durum wheat, a strategic crop in Algeria and throughout the Mediterranean region, allows the evaluation of the impact of bioinoculants in a real agricultural context [24], especially as the bacterial strains used in this study were originally isolated from its rhizosphere, thus reinforcing their suitability for this culture.

In this context, the general objective of this study was to develop alginate capsules loaded with two plant probiotic agents (*Bacillus thuringiensis* strain B25 and *Pantoea agglomerans* strain Pa), ensuring prolonged viability, preservation of PGP activities, and controlled delivery of strains. The encapsulation efficiency, survival rate, release kinetics, colonization capacity of *Arabidopsis thaliana* in a hydroponic medium, and the effect of encapsulated bacteria on the growth of durum wheat were studied.

## 2. Materials and methods

### 2.1. Bacterial strains

The two bacterial strains used in this study were *Pantoea agglomerans* strain Pa and *Bacillus thuringiensis* strain B25. Both strains were selected from a previous study [25]. The Pa strain was isolated from the rhizosphere of durum wheat fields in the Bou-Saâda region, Algeria, while B25 was isolated from the rhizosphere of durum wheat in the North Sétif region, Algeria. Both strains were previously tested for their plant growth-promoting activities and for the absence of antagonism between them [25].

### 2.2. Bacterial growth under drought stress

Luria-Bertani broth (LB) medium was prepared by adding polyethylene glycol (PEG_6000_) at different concentrations (0%, 10%, 20%, 30%, 40%, and 50%) and inoculating with 1% pre-cultures of the two bacterial strains [24-h cultures with OD = 1.4, 1.99 × 10^8^ (±0.11 × 10^8^) CFU/mL for B25, and OD = 0.78, 2.22 × 10^8^ (±0.11 × 10^8^) CFU/mL for Pa]. After incubation at 30 °C with shaking (200 rpm) for 48 h, growth was estimated by measuring optical density at 600 nm after 24 and 48 h using a spectrophotometer. The growth of the two bacterial strains at different PEG_6000_ concentrations was measured. The direct relationship between water potential and its growth inhibitory effect was deduced.

### 2.3. Effect of osmoprotectants on bacterial survival

In order to test the effect of osmoprotectants on the growth and survival of the two tested strains, LB medium with 50% PEG_6000_ was supplemented with different osmoprotectants (glycine, betaine, choline, glutamate, mannitol, and proline). Osmoprotectants are added to the medium at a final concentration of 5 mM. Media inoculated with 1% pre-culture were incubated with shaking (200 rpm) at 30 °C for 48 h. Growth was measured at 600 nm using a spectrophotometer.

### 2.4. Microorganisms and culture conditions

Bacteria, previously preserved in 15% glycerol at −20 °C, were cultured in 500 mL of sterile LB medium adjusted to pH 7. Cultures were incubated with shaking (200 rpm) at 30 °C to harvest the cells after 48 h of incubation. Cultures were then centrifuged at 3000 rpm for 30 min. The pellet of each strain was suspended in 10 mL of tryptone-salt broth. For co-inoculation, an individual inoculum of each strain was prepared and centrifuged, and the two pellets were jointly resuspended in 10 mL of tryptone-salt broth. Bacterial concentrations in the final tryptone-salt solution were estimated at 8.15 × 10^10^ (±0.01 × 10^10^) CFU/mL for Pa and 1.42 × 10^9^ (±0.32 × 10^9^) CFU/mL for B25. The bacterial mixture included 9.17 × 10^7^ (±0.01 × 10^7^) CFU/mL of Pa and 8.48 × 10^8^ (±0.33 × 10^8^) CFU/mL of B25. The dilution plating technique on LB agar plates at 30°C for 24 h was used for the estimation of bacterial concentration.

### 2.5. Cell bioencapsulation

The extrusion method (ionic gelation) was used as a bioformulation technique following the method described by Wu et al. [26]. The matrix solution was prepared by mixing 1% alginic acid (A-7128 Sigma, Steinheim, Germany; high viscosity (14,000 cps at 2%)) and proline 5mM in distilled water. Then, 10 mL of the previously prepared bacterial suspensions were mixed with 100 mL of sterile matrix solution and stirred for 30 min to obtain a homogeneous solution. The mixture was transferred into a sterile syringe and placed on a sterile CaCl_2_ solution (2%) with stirring (150 rpm). After 30 min of contact with the calcium solution, the gelled beads were recovered, washed 5–6 times with sterile distilled water, placed on filter paper in a Petri dish, and dried sterilely under flow for 24 h at room temperature. The dried beads were stored at 4 °C until further use.

### 2.6. Encapsulation efficiency (EE)

The encapsulation efficiency (EE) of bacteria was evaluated using Eq 1 according to Panichikkal et al. [27]:

$$\text{EE}=\left({\text{N}}_{0}-\text{Ne}\right)/{\text{N}}_{0}\times \text{1}00$$ (1)

Where N_0_ is the number of bacteria contained in the alginate solution, and Ne represents the number of bacteria present in the CaCl_2_ solution.

Bacterial enumeration on nutrient agar (NA) from different dilutions was used to determine the number of bacteria in both solutions. All plated nutrient agar plates were incubated at 30 °C for 24 h.

### 2.7. Survival rate

The survival rate of bacteria during the encapsulation process was determined according to Chi et al. [28] by dividing the number of bacteria contained in the dried beads (N) by the number of bacteria contained in the fresh beads (N_0_) according to Eq 2 and expressed as a percentage:

Survival rate=(N/N0)×100 (2)

### 2.8. Verification of cell viability and PGP activities during storage

Every month, 1 g of dried beads, stored at 4 °C, was collected and dissolved in 10 mL of 0.1 M phosphate buffer (pH 7.0) at 30 °C for 1 h in a rotary shaker at 200 rpm. These beads were then sterilely ground in the same solution, and the released bacteria were counted using the conventional nutrient agar (NA) plate enumeration method. Briefly, a series of dilutions of each bioformulation was prepared in 0.85% sterile saline solution; then, 0.1 mL of each dilution was plated on NA plates. Colony forming units (CFU/g) were calculated after incubation at 30 °C for 24 h.

To analyze the production of indole acetic acid (IAA) by the bacteria trapped in the dried capsules, 100 µL of the ground material of each bioformulation was inoculated into 10 mL of nutrient broth supplemented with 0.2% L-tryptophan; the tubes were incubated at 30 °C for 4 days. After incubation, the supernatant was collected by centrifugation at 12,000 rpm for 10 min, and 0.5 mL of supernatant was mixed with 1 mL of Salkowski reagent. Then, the tubes were kept in the dark for 30 min. The development of a red color indicated IAA production and the absorbance was read at 530 nm. The quantity of IAA (µg/mL) was calculated according to a previously determined pure IAA calibration curve [29].

The production of siderophores was assessed by the Chrome Azurol S (CAS) medium according to Saidi et al. [30]. 10 mL of iron-restricted King B medium was inoculated with 100 µL of the ground material from each bioformulation and incubated at 30 °C for 4 days. After centrifugation of the cultures at 12,000 rpm for 10 min, 500 µL of the CAS reagent was added to 500 µL of the supernatant. The mixture was then incubated in the dark for 30 min. A color change from blue to orange indicated the production of siderophores, and their production was read at 630 nm. The percentage of siderophore units was determined according to Eq 3:

Discoloration (%)=[(Ar×As)/Ar] 100 (3)

Where Ar is the absorbance of the control, and As is the absorbance of the sample.

The quantitative estimation of phosphate solubilization by encapsulated bacteria was determined on liquid Pikovskaya (PVK) medium, containing insoluble phosphate. 10 mL of liquid PVK were inoculated with 100 µL of grounded bacterial homogenate and incubated at 30 °C for 7 days. Cultures were harvested by centrifugation at 12,000 rpm for 10 min, and in-culture phosphorus was estimated using the method described by Slama et al. [31]. 500 µL of supernatant was mixed with 500 µL of 10% (w/v) trichloroacetic acid in a test tube to which 4 mL of color reagent was added (1:1:1:2 ratio of 3M H_2_SO_4_, 2.5% (w/v) ammonium molybdate, 10% (w/v) ascorbic acid, and distilled water) and incubated at room temperature for 30 min. The absorbance of blue color was measured at 630 nm. The amount of soluble phosphate was detected from the standard curve of KH_2_PO_4_.

### 2.9. Swelling properties

The study of capsule swelling was carried out according to Wu et al. [26]. 1 g of dried beads was immersed in a solution of 10 mL of 0.85% NaCl for 10 days. Swollen beads were removed each day, and excess solution was removed by gently pressing the beads between two pieces of absorbent paper. The inflated beads were then weighed using an electronic precision balance. The swelling rate was estimated according to Eq 4:

Swelling ratio (SR)=Ws/Wd×100 (4)

Where Ws is the weight of the inflated beads at time t, and Wd is the weight of the initial dry beads at t0. All experiments were carried out in triplicate.

### 2.10. Release properties

The cumulative release of viable bacterial cells released from the bioformulated capsules was measured by immersing 1 g of capsules from the stock after 6 months of storage at 4 °C in 10 mL of PBS (pH 7.2) and incubating them for 60 days at room temperature. Volumes were taken at different intervals, and the cell number in the collected solution was determined by the counting method on nutrient agar (NA) plates. All experiments were carried out in triplicate.

### 2.11. Arabidopsis thaliana test

#### 2.11.1. Growing conditions

In order to evaluate the effect of root colonization of free and encapsulated tested bacteria on the growth and development of *Arabidopsis thaliana*, the seeds of the wild-type *Arabidopsis thaliana* Columbia Col-0 obtained from Lehle Seeds (USA) were surface sterilized first for 1 min in 70% ethanol and then in sodium hypochlorite (1%, 5 min) and rinsed (6 times) with sterile distilled water. For germination on agar medium, seeds were sown on the surface of ½ Murashige and Skoog (MS) medium (pH 5.7) devoid of sucrose and supplemented with 0.8% (w/v) agar. All plates were covered and sealed with Parafilm paper and placed in a germination chamber maintained at 22  ±  2 °C with a 16/8 h light/dark photoperiod. Four days after germination, 10 seedlings were transferred to half of a Petri dish containing MS (½). For free cultures, each bacterium was inoculated individually with a cell concentration of 1 × 10^8^ (±0.2 × 10^8^) CFU/mL in the opposite half of the Petri dish, while 50 dried beads were placed in the opposite half of the Petri dish for each of the encapsulated bioformulations.

Each treatment included five replicates with a total of 50 seedlings. The plates were incubated for 11 days under the same germination conditions. At the end of this period, root length and root and leaf biomass were recorded.

The ability of free and encapsulated strains to attach and colonize seedling roots was estimated visually using the triphenyltetrazolium chloride (TTC) procedure [32], while ImageJ software was used to analyze the leaf surface.

The plates were divided into five groups representing the five treatments, as follows: (1) uninoculated seedlings (negative control); (2) seedlings inoculated with free B25 (B25 F); (3) seedlings inoculated with encapsulated B25 (B25 E); (4) seedlings inoculated with free Pa (Pa F); and (5) seedlings inoculated with encapsulated Pa (Pa E).

#### 2.11.2. Chlorophyll and carotenoid estimation

Chlorophylls a, b, and total and carotenoid estimation were carried out according to Kerbab et al. [29]. 0.5 g of leaves from each sample was cut into small segments (0.5 cm), homogenized in 10 mL of 80% acetone, and stored at −10 °C overnight. The organic extract was centrifuged at 14,000 rpm/5 min, and the absorbance of the supernatant was measured by spectrophotometer at 663 nm for chlorophylls a (chla) (Eq 5), at 645 nm for chlorophylls b (chlb) (Eq 6), total chlorophyll (Chla+b) (Eq 7), and at 470 nm to determine carotenoids (Eq 8).

Chla=12.70 A663−2.69 A645 (5)

Chlb=22.90 A645−4.68 A663 (6)

Chla+b=20.21 A645+8.02 A663 (7)

Carotenoids=(1000 A470−1.9 Chla−63.14 Chlb)/214 (8)

### 2.12. Effects of application of free and encapsulated rhizobacteria on durum wheat growth

#### 2.12.1. Disinfection and germination of seeds

Wheat seeds of the Bousselam variety (*Triticum durum* L.c.v Bousselam) (Pedigree: Heider/Marli/Heider-Cro ICD-414-1BLCTR-4AP) were surface-disinfected with ethanol (70% for 1 min) and then with sodium hypochlorite (1% for 30 min) and rinsed several times with sterile distilled water. Sterilized seeds were germinated on filter paper in Petri dishes containing 10 mL of sterile distilled water at 22 °C for 48 h in the dark.

#### 2.12.2. Preparation of free bacterial inoculums

Both strains were inoculated into LB broth with constant stirring at 150 rpm for 48 h at 30 °C. Cultures were centrifuged at 3000 rpm for 30 min. The pellet of each strain was suspended in 0.85% sterile saline and adjusted to 1 × 10^8^ (±0.2 × 10^8^) CFU/mL.

#### 2.12.3. Sowing and seed growth

Plastic pots (9 cm in diameter and 10 cm in height) were disinfected with a sodium hypochlorite solution and filled with 550 g of sand sterilized at 180 °C for 1 h for 3 alternate days. These pots were watered with 10% (v/w) of a ½ Hoagland solution before sowing. For samples inoculated with free strains, sowing of seeds was carried out by taking germinated seeds (3 mm radicle), sowing them (5 seeds/pot) at a depth of 1 cm from the surface in each pot, and inoculating with 1 mL/pot of suspension of a fresh culture prepared as described previously. For encapsulated samples, 1 g of capsules containing 3.05 × 10^7^ (±0.15 × 10^7^) CFU/g for B25 and 5.7 × 10^6^ (±0.04 × 10^6^) CFU/g for Pa were removed from the stock after 6 months of storage at 4 °C and placed at a depth of 1 cm near the sown seeds. The pots were divided into five groups representing the five treatments: (1) uninoculated seeds (negative control); (2) seeds inoculated with free B25 (B25 F); (3) seeds inoculated with encapsulated B25 (B25 E); (4) seeds inoculated with free Pa (Pa F); and (5) seeds inoculated with encapsulated Pa (Pa E).

The pots were kept for 45 days in a growth chamber at an average day/night temperature of 25 and 16 °C, respectively, and a light photoperiod of 16 h. The humidity of the pots was controlled by periodic watering with constant doses of sterile water [33].

Plants were harvested after 45 days, roots were washed with distilled water, and shoots were separated. Morphological (shoot and root lengths, fresh and dry weights) and biochemical parameters (chlorophyll pigments, total sugars, and total proteins) were analyzed. Bacterial survival was determined by analyzing the rhizospheric and root colonization ability of the inoculated bacteria. During treatment, plants were removed at two different time points (after 15 and 30 days) before the final harvest for the analysis of morphological parameters and rhizospheric, epiphytic, and endophytic bacterial enumerations.

#### 2.12.4. Evaluation of morphological parameters

Shoot and root lengths (cm) and fresh and dry weight of shoots and roots (g) were measured. Morphological measurements were carried out in triplicate.

#### 2.12.5. Dosage of chlorophylls and carotenoids

The contents of chlorophyll a, b, total, and carotenoids were determined in triplicate, as described previously.

#### 2.12.6. Total sugar estimation

The extraction of total sugars from fresh leaf and root material was carried out according to the following protocol: 3 mL of ethanol (80%) was added to 0.1 g of leaves. The mixture was incubated at room temperature in the dark for 48 h. Then, the mixture was heated to 80 °C in a water bath to evaporate the ethanol, and then 20 mL of distilled water was added to the mixture. Total sugars were determined according to the method of Dubois et al. [34]. The reaction mixture contained 0.5 mL of the sample, 0.5 mL of a phenol solution (5%), and 2.5 mL of 12 M sulfuric acid. The color intensity proportional to the sugar concentration was measured by spectrophotometer at OD 490 nm. The values obtained were translated into glucose concentrations in reference to a previously established calibration curve. All biochemical parameters were carried out in triplicate.

#### 2.12.7. Total protein estimation

Soluble protein content was estimated by grinding 0.25 g of the fresh tissue samples (shoots and roots) in liquid nitrogen. The resulting powder was solubilized in 5 mL of 0.1 M potassium phosphate buffer, pH 7.0. After centrifugation of the homogenate at 12,000 rpm for 15 min at 4 °C, the concentration of supernatant soluble proteins was determined spectrophotometrically according to Lowry et al. [35].

#### 2.12.8. Enumeration of rhizospheric bacteria

1 g of rhizospheric sand was homogenized in 10 mL of sterile physiological water and stirred for 10 min. 100 µL of the sample and of each decimal dilution ranging from 10^−1^ to 10^−6^ was spread in duplicate on nutrient agar and incubated at 30 °C for 48 h. Bacterial counts were expressed in CFU/g of soil.

#### 2.12.9. Enumeration of root epiphytic bacteria

1 g of roots from each treatment was washed three times with sterile distilled water in order to remove all attached sand; roots were then transferred into 10 mL of sterile physiological water and stirred for 15 min. Dilutions ranging from 10^−1^ to 10^−6^ were prepared, and the number of epiphytes attached to the roots was determined by spreading 100 µL of each dilution on nutrient agar medium. Incubation was carried out at 30 °C for 48 h, and bacterial counts were expressed in CFU/g of roots.

#### 2.12.10. Enumeration of root endophytic bacteria

The root surface was first disinfected by immersing the roots in 70% ethanol for 1 min and then in 1% sodium hypochlorite for 20 min and rinsing several times with sterile distilled water. To check the effectiveness of root disinfection, the final wash water was spread on nutrient agar, and plates were incubated at 30 °C for 48 h. 1 g of disinfected roots was crushed and homogenized in 10 mL of sterile physiological water. 100 µL serial dilutions up to 10^−6^ of the samples were spread on the surface of NA medium and incubated at 30 °C for 48 h. Bacterial counts were expressed in CFU/g of roots.

### 2.13. Statistical analysis

Statistical analysis of the data was performed using analysis of variance (ANOVA). When significant effects were detected, groups were compared using a post-hoc Tukey's HSD test. The significance level used for all statistical tests was 5% (p < 0.05). The statistical programs used in the analysis were GraphPad Prism 9.4.0 and OriginPro 2022.

## 3. Results

### 3.1. Response of bacterial strains to drought stress and osmoprotectants addition

In order to evaluate which level of water stress affected bacterial growth, strains were cultured in liquid LB medium supplemented with different concentrations of PEG_6000_ (0%, 10%, 20%, 30%, 40%, and 50%). The PEG presence produced a significant effect on growth after 48 h of incubation (Figure S1A), with 50% concentration inducing a maximum decline in the growth of the two tested strains. Therefore, this concentration was selected for the osmoprotectant application study. Both strains showed the same behavior in response to drought stress. The application of osmoprotectants showed that no significant improvement in growth was recorded with the exception of mannitol for B25 and proline for the two strains (Figure S1B); the addition of both compounds allowed considerably higher cell density in 48h, up to an OD value of 0.27 and 0.19 compared to 0.145 and 0.158 in the control for B25 and Pa, respectively.

### 3.2. Bioencapsulation

**Figure 1.**
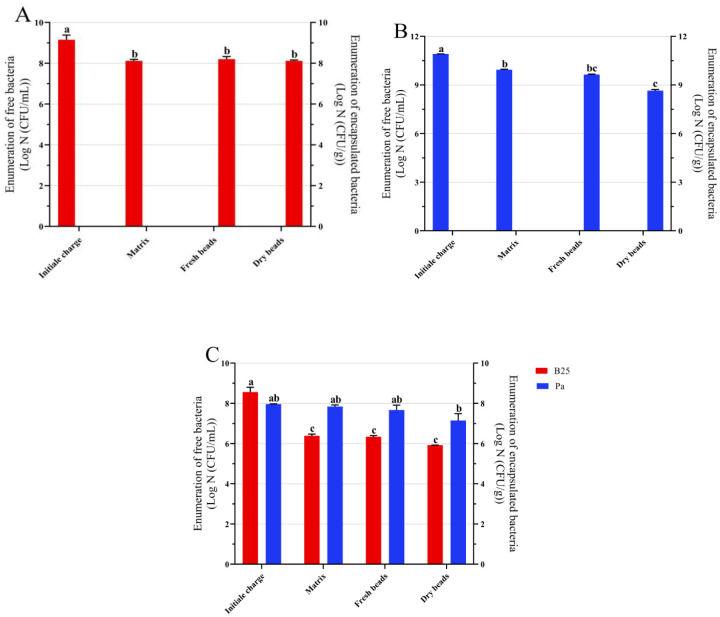
Survival of (A) B25, (B) Pa, and (C) B25+Pa at different stages of the bioencapsulation process. Bar plots represent the average standard error of three different experiments. GraphPad Prism9.4.0 was used to perform statistical analysis using two-way ANOVA and Tukey's multiple comparison post-hoc test. The CFUs corresponding to each treatment and not sharing the same letters are significantly different according to Tukey's HSD post-hoc test.

The formed beads after gelation had a spherical shape whose diameter varies between 2.48 (±0.18) and 3.17 (±0.18) mm, while the largest diameter after drying was attributed to B25 beads (1.11 ± 0.06 mm) (Table 1). The total number of viable cells was calculated at each step of the encapsulation process. The starting cell concentration of B25 was 1.42 × 10^9^ (±0.32 × 10^9^) CFU/mL and that of Pa was 8.15 × 10^10^ (±0.01 × 10^10^) CFU/mL; a concentration of 3.66×10^8^ (±0.33 × 10^8^) CFU/mL for B25 and 9.18 × 10^7^ (±0.01 × 10^7^) CFU/mL for Pa was added to the alginate matrix solution for their mixture (Figure 1A–C). After bioencapsulation, cell viability was not significantly modified for B25 when encapsulated alone or in mixture; this resulted in a survival rate of 98.60% after drying (Table 1). The number of bacteria in the dried encapsulated Pa beads was found to be 4.48 × 10^8^ (±0.09 × 10^8^) CFU/g and 1.407 × 10^7^ (±0.34 × 10^7^) CFU/g when encapsulated alone and in combination, respectively (Figure 1A–C). These results correspond to an excellent encapsulation efficiency (EE) where more than 99% of cells were effectively trapped in the gel capsules for all treatments.

**Table 1.** Characteristics of the different alginate beads.

Treatment	Fresh weight (mg)	Dry weight(mg)	Fresh beads size (mm)	Dry beads size (mm)	Survival rate (%)	Entrapment efficiency (EE) (%)
B25	17.18 ± 1.17	0.24 ± 0.05	3.17 ± 0.18	1.11 ± 0.06	98.60	99.97
Pa	12.64 ± 0.68	0.55 ± 0.07	2.61 ± 0.1	1.06 ± 0.08	89.68	99.92
B25+Pa	17.84 ± 1.21	0.51 ± 0.06	2.48 ± 0.18	1.08 ± 0.04	93.23	99.92

### 3.3. Bead storage studies

#### 3.3.1. Survival of encapsulated strains

**Figure 2.**
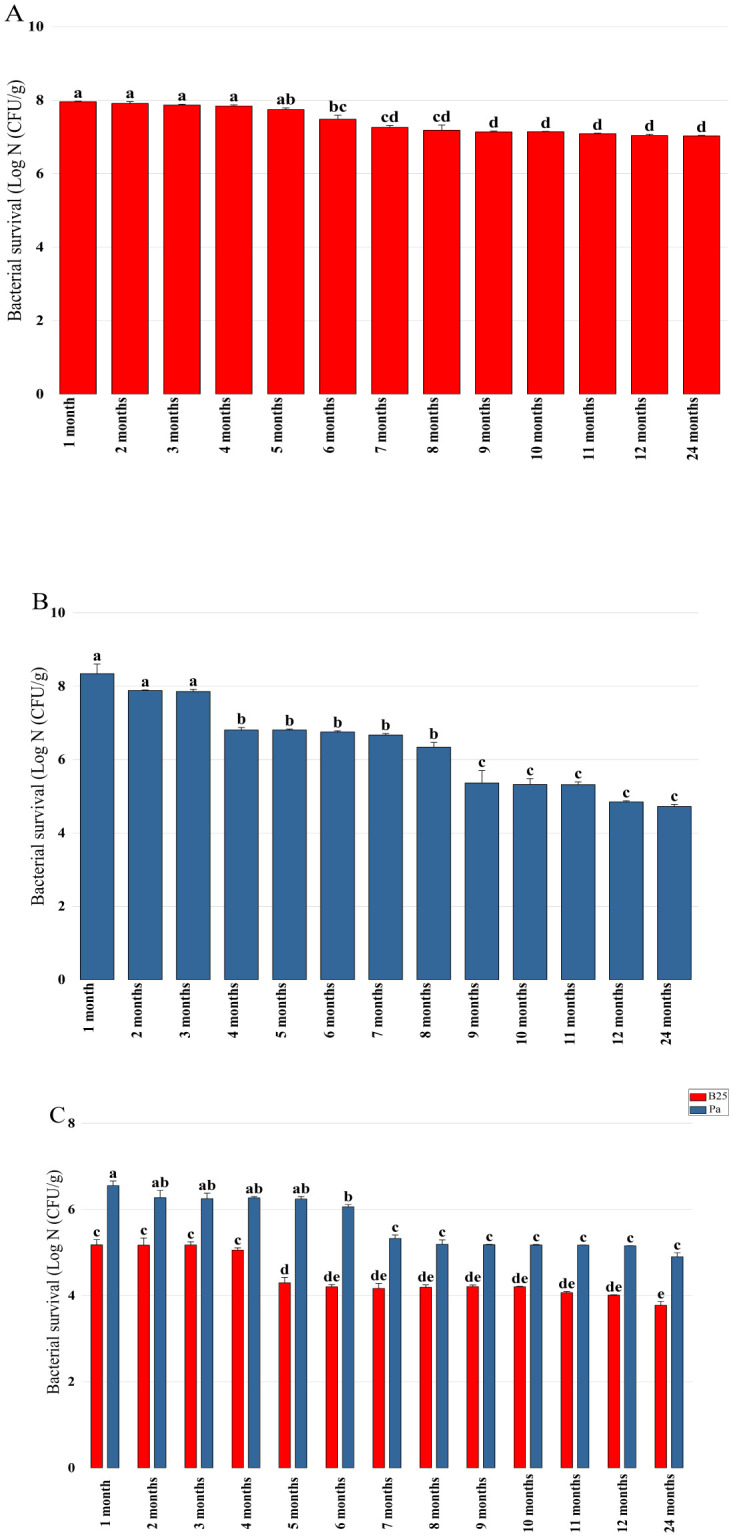
Survival of (A) B25, (B) Pa, and (C) B25+Pa during 24 months of storage. Bar plots represent the average standard error of three different experiments. OriginPro2022 was used to perform statistical analysis, using two-way ANOVA and Tukey's multiple comparison post-hoc test. CFUs corresponding to each treatment and not sharing the same letters are significantly different according to Tukey's HSD post-hoc test.

Survival analysis was carried out every month after storing the dried beads at 4 °C for 24 months. The number of viable cells in B25 beads remained almost constant during the 24 months of storage (Figure 2A). Pa beads showed a lower survival rate: the number of viable cells reached 7 × 10^4^ (±0.04 × 10^4^) CFU/g after 12 months and 5.3 × 10^4^ (±0.07 × 10^4^) CFU/g after 24 months of storage (Figure 2B). For bacterial mixture beads, the bacterial load of the two strains decreased by one log during the first storage phase (from 1 month to 6 months) and then stabilized and reached 5.9 × 10^3^ (±0.11 × 10^3^) CFU/g for B25 and 8×10^4^ (±0.13 × 10^4^) CFU/g for Pa after 24 months of storage (Figure 2C). The survival parameter can be useful in the selection of bioformulations for crop inoculation.

#### 3.3.2. Maintenance of PGP traits by encapsulated strains over time

**Figure 3.**
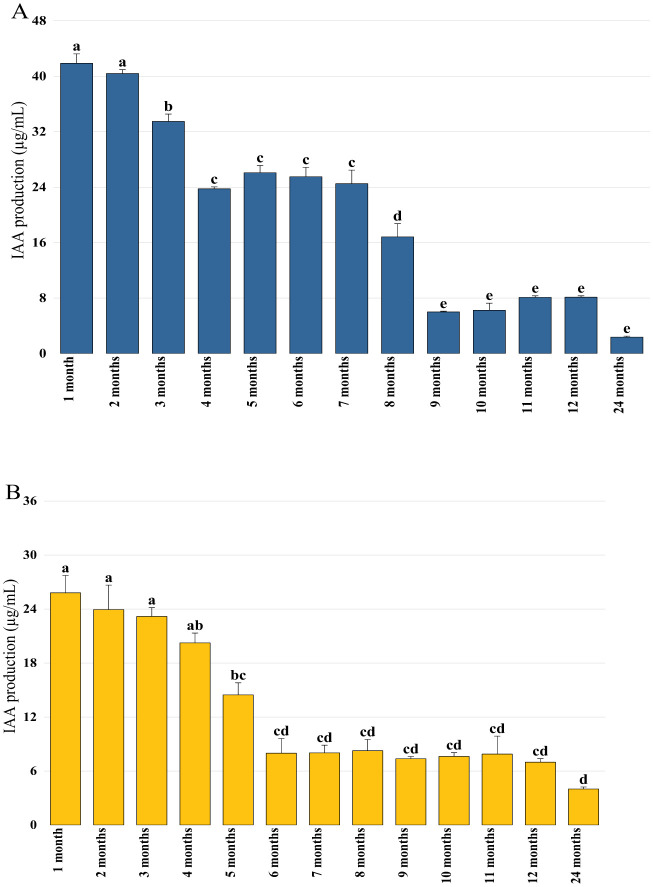
IAA production by (A) Pa and (B) B25+Pa during 24 months of storage. Bar plots represent the average standard error of three different experiments. OriginPro2022 was used to perform statistical analysis, using two-way ANOVA and Tukey's multiple comparison post-hoc test. The quantities of IAA corresponding to each treatment and not sharing the same letters are significantly different according to Tukey's HSD post-hoc test.

**Figure 4.**
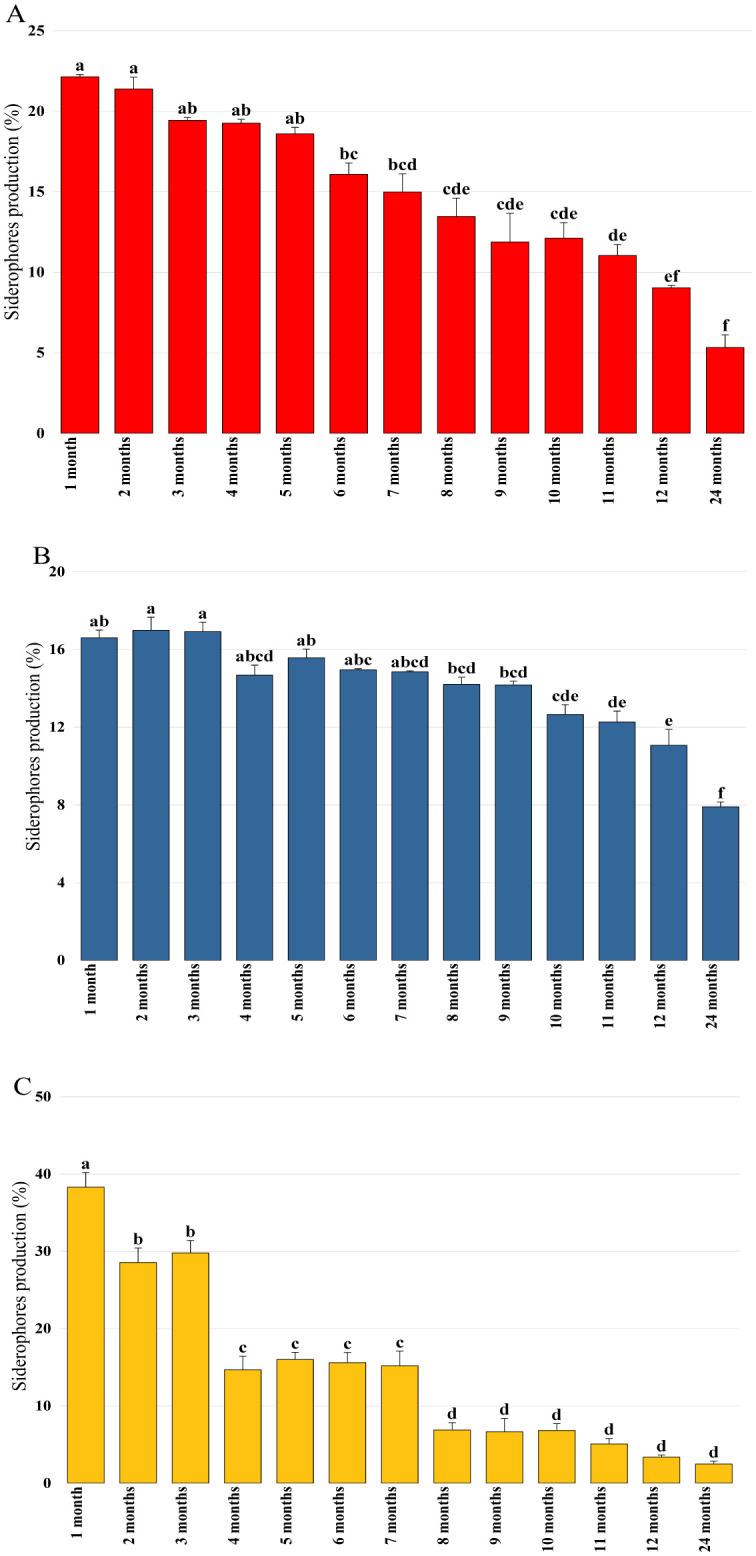
Siderophores production by (A) B25, (B) Pa, and (C) B25+Pa during 24 months of storage. Bar plots represent the average standard error of three different experiments. OriginPro2022 was used to perform statistical analysis, using two-way ANOVA and Tukey's multiple comparison post-hoc test. The quantities of siderophores corresponding to each treatment and not sharing the same letters are significantly different according to Tukey's post-hoc HSD test.

**Figure 5.**
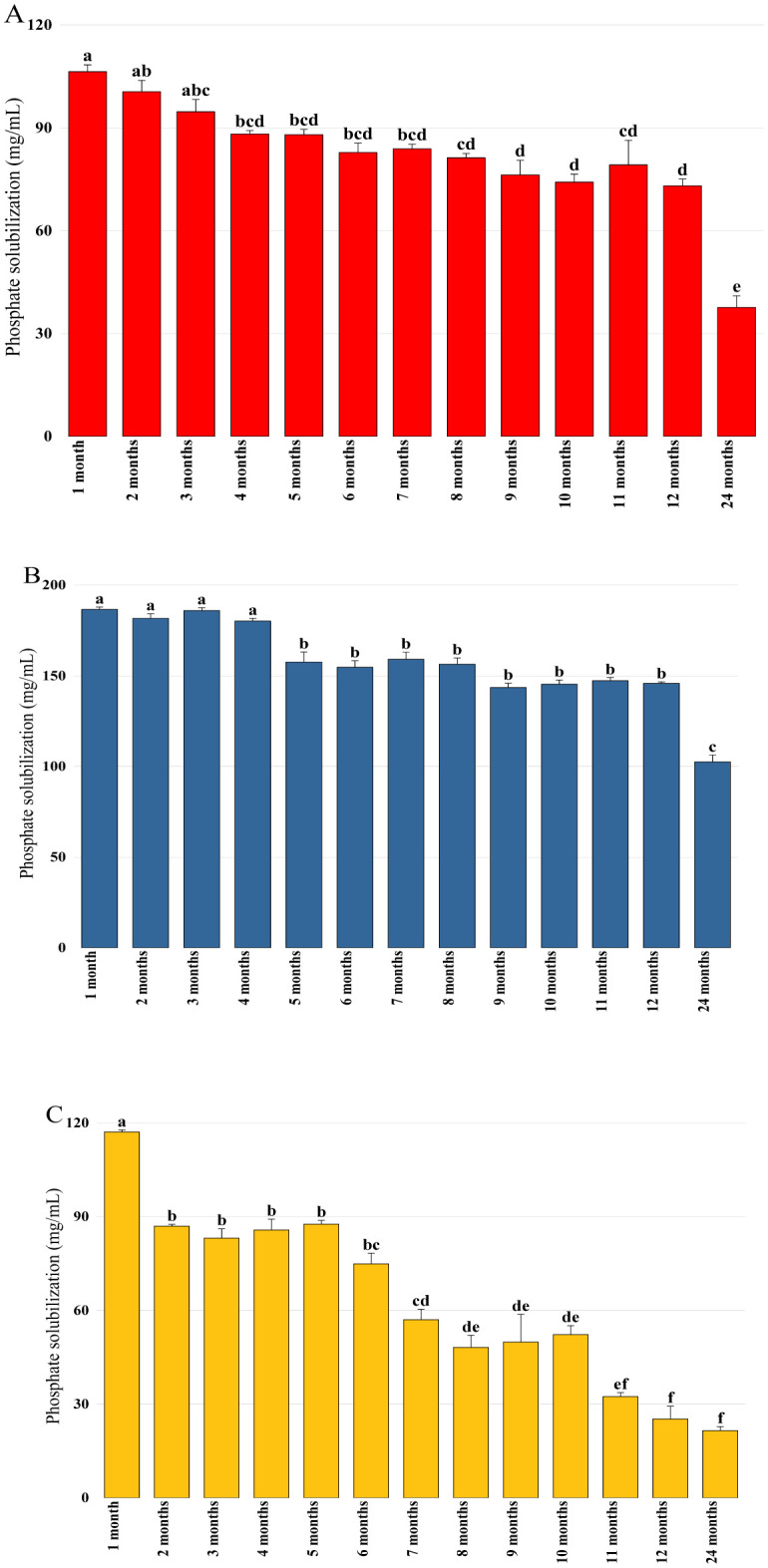
Phosphate solubilization by (A) B25, (B) Pa, and (C) B25+Pa during 24 months of storage. Bar plots represent the average standard error of three different experiments. OriginPro2022 was used to perform statistical analysis, using two-way ANOVA and Tukey's multiple comparison post-hoc test. The quantities of phosphate corresponding to each treatment and not sharing the same letters are significantly different according to Tukey's HSD post-hoc test.

In order to verify the conservation of plant growth-promoting properties during storage, activities linked to phytostimulation (IAA production), biocontrol (siderophore production), and biofertilization (phosphate solubilization) were quantified each month. The obtained results confirmed that Pa retained its capacity to produce IAA in its encapsulated state, where its production proved to be stable during the first three months and reached 18.88 µg/mL after eight months of storage (Figure 3A). IAA production by the bacterial mixture beads experienced a significant initial decline after the first month and reached 25.8 µg/mL. A second decline was recorded after six months when the production was 7.98 µg/mL. After this decline, IAA production by Pa remained low and constant for up to 24 months of storage (Figure 3B). Siderophore production was detected in all bioformulations at varying levels. A stable and significant activity was observed by the Pa beads even after 12 months of storage (Figure 4B), while a progressive decrease in production was recorded by encapsulated B25, which reached 9.04% after one year of storage (Figure 4A). Bacterial mixture siderophore production reached activity levels of 15.6%, 3.36%, and 2.49% after 6, 12, and 24 months of storage, respectively (Figure 4C). Phosphate solubilization was found to be high in the case of Pa bioformulation, followed by B25 and their mixture. The statistical study revealed that phosphate solubilization remained stable by Pa, which reached 102.58 mg/mL after 24 months (Figure 5B). For B25, the quantity of phosphate remained constant from the first month to 12 months of storage, where it was found to be 73.09 mg/mL (Figure 5A). A notable decrease in solubilized phosphate over time by the encapsulated mixture was recorded, where an amount of 21.5 mg/mL was detected after 24 months of storage (Figure 5C).

### 3.4. Swelling study and release kinetics

**Figure 6.**
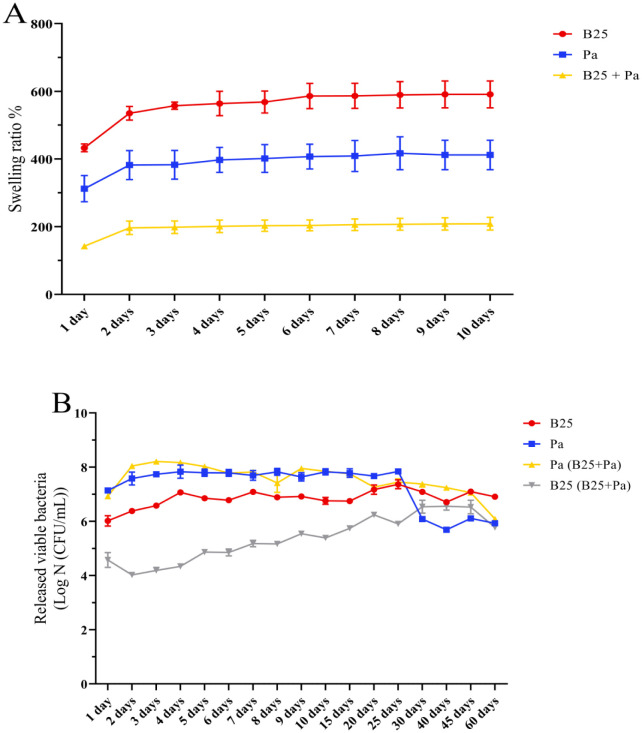
(A) Swelling rate (%) of dry capsules of B25, Pa, and B25+Pa and (B) release kinetics of bacteria from dry capsules of B25, Pa, and B25+Pa. GraphPad Prism9.4.0 was used to perform statistical analysis, using a correlation of the means from three different experiments.

The swelling rate was studied at room temperature for 10 days. The capsules of all formulations swelled rapidly on the first day; a gradual but negligible increase was noted from the second to the eighth day of the study. Swelling equilibrium was reached after the ninth day where rates of 590.98%, 412.06%, and 209% were recorded for the B25 and Pa capsules and their combination, respectively (Figure 6A). The number of released cumulative bacteria by the dry B25 capsules was significantly higher on the seventh day compared to the first day (Figure 6B). This number also increased after 25 days and reached 8.1 × 10^6^ (±0.11 × 10^6^) CFU/mL on the 60th day of the experiment. The release kinetics of Pa was divided into two phases: the first, between the 1st and the 25th day, was regular and high, with the number of viable released and accumulated bacteria varying between 1.38 × 10^7^ (±0.1 × 10^7^) CFU/mL and 7 × 10^7^ (±0.06 × 10^7^) CFU/mL. The second phase was short and experienced a rapid decline on the 30th day, from where the release rate decreased and reached 8.5 × 10^5^ (±0.03 × 10^5^) CFU/mL on the 60th day. Regarding the bacterial mixture, the release rate of Pa was significantly higher than that of B25. The number of live B25 cells released from the capsules increased steadily with time and reached the highest release equilibrium of 3.4 × 10^6^ (±0.33 × 10^6^) CFU/mL after 30 days (Figure 6B). The release of Pa progressively declined from the 5th day, reaching the lowest release rate of 1.2 × 10^6^ (±0.02 × 10^6^) CFU/mL after 60 days (Figure 6B). The two strains released from the bacterial mixture beads were distinguished on the agar plate by their distinctive phenotypes: Pa forms yellow, round, regular colonies around 3 mm in diameter and smooth in texture, while B25 is characterized by larger colonies of approximately 8–12 mm in diameter, slightly irregular in shape, white in color, and powdery in texture.

### 3.5. Inoculation test on *Arabidopsis thaliana*: effects on morphological, colonization, and biochemical parameters

#### 3.5.1. Morphological parameters and leaf area analysis

**Figure 7.**
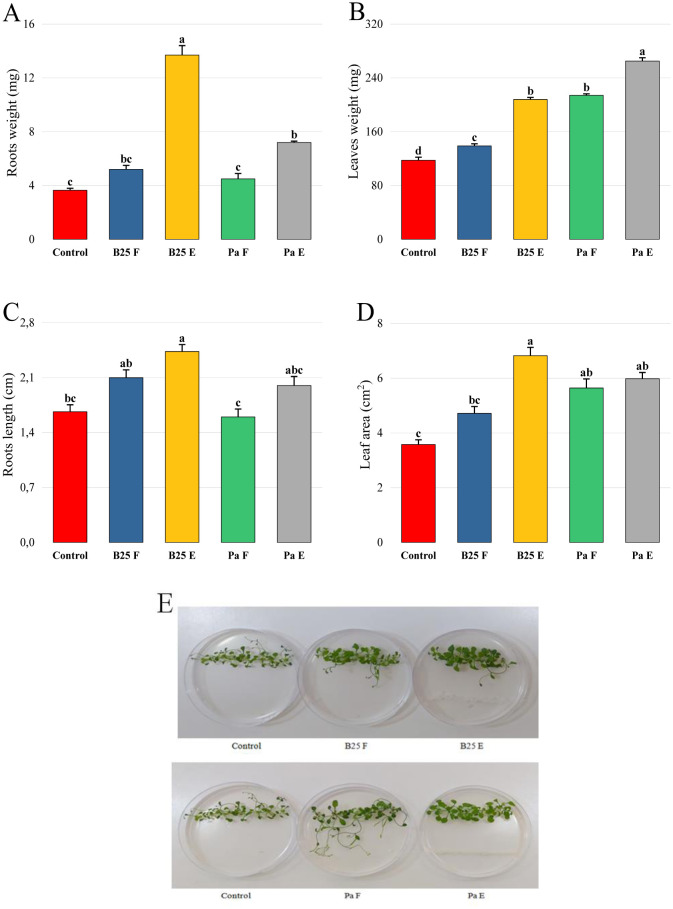
Effect of free (F) and encapsulated (E) bacteria inoculation on (A, B) fresh weight of roots and shoots (g), (C) roots length (cm), (D) leaf surface, and (E) rosette color of *Arabidopsis thaliana* seedlings grown on ½ MS medium. Bar plots represent the mean ± standard error of three experiments. OriginPro2022 was used to perform statistical analysis, using two-way ANOVA and Tukey's multiple comparison post-hoc test. The weight, length, and leaf area values corresponding to each treatment and not sharing the same letters are significantly different according to Tukey's HSD post-hoc test.

To evaluate the effect of inoculation of the two tested bacterial strains on the growth of *Arabidopsis thaliana* seedlings, Petri dishes supplied with ½ MS medium were used. Our results show that the two strains used in the free and encapsulated form are capable of stimulating the length of the root system and the production of plant biomass (Figure 7A–C). B25 beads increased root fresh weight by three times compared to non-inoculated seedlings (Figure 7A). A significant increase in leaf weight was observed in seedlings inoculated with encapsulated B25 and Pa compared to the same treatments freely inoculated (Figure 7B). Leaf area analysis revealed that this parameter increased significantly in seedlings inoculated with B25 and Pa compared to non-inoculated seedlings (Figure 7D), indicating that the inoculated plants produced larger-sized leaves than the control plants. A more pronounced increase was noted in seedlings inoculated with encapsulated B25, whose leaf area was 91% higher than that of control seedlings (Figure 7D). Thus, a visual analysis with the naked eye of the Ms plates allowed us to observe a more accentuated green color of the rosettes of inoculated treatments compared to that of non-inoculated control (Figure 7E).

The revelation of *Arabidopsis thaliana* root colonization by free and encapsulated rhizobacteria was carried out aseptically using triphenyltetrazolium chloride (TTC), and roots were photographed (Figure S2). Qualitative root colonization of the inoculated seedlings was noted by a significant difference between the roots of the free and encapsulated inocula, which were more colorful. This strongly suggests that the level of attraction between roots and bacteria is greater in encapsulated strains and that adhesion to roots is stronger.

#### 3.5.2. Dosage of chlorophyll pigments

**Figure 8.**
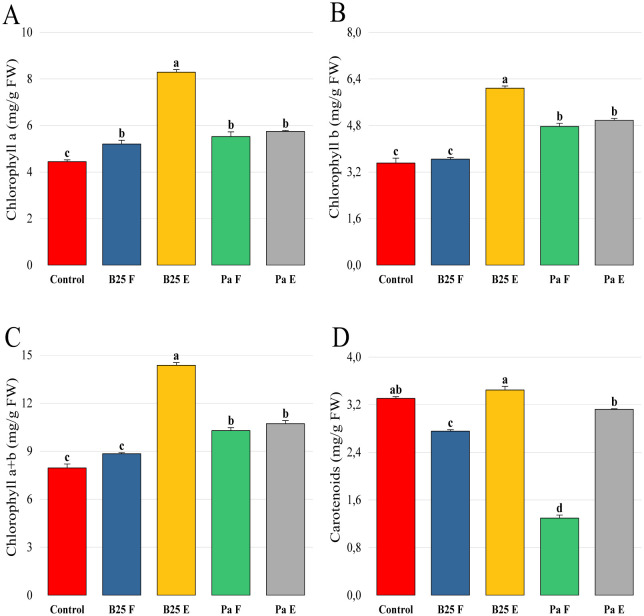
Effect of free and encapsulated bacterial inoculation on the content of (A) chlorophyll a (mg/g FW), (B) chlorophyll b (mg/g FW), (C) chlorophyll a + b (mg/g FW), and (D) carotenoids (mg/g FW) of *Arabidopsis thaliana* seedlings grown on ½ MS medium. Bar plots represent the mean ± standard error of three experiments. OriginPro2022 was used to perform statistical analysis, using two-way ANOVA and Tukey's multiple comparison post-hoc test. The values corresponding to each treatment and not sharing the same letters are significantly different according to Tukey's HSD post-hoc test.

The application of B25 and Pa beads on *Arabidopsis thaliana* showed a significant increase in the content of chlorophyll pigments. Chlorophylls a, b, and total were significantly improved in plants inoculated with encapsulated B25 and Pa, while treatment with free strains had a non-significant effect on carotenoid content, whose levels were lower than control levels (Figure 8A–D).

### 3.6. Potential of encapsulated bacterial strains on durum wheat growth

#### 3.6.1. Morphological parameters

**Figure 9.**
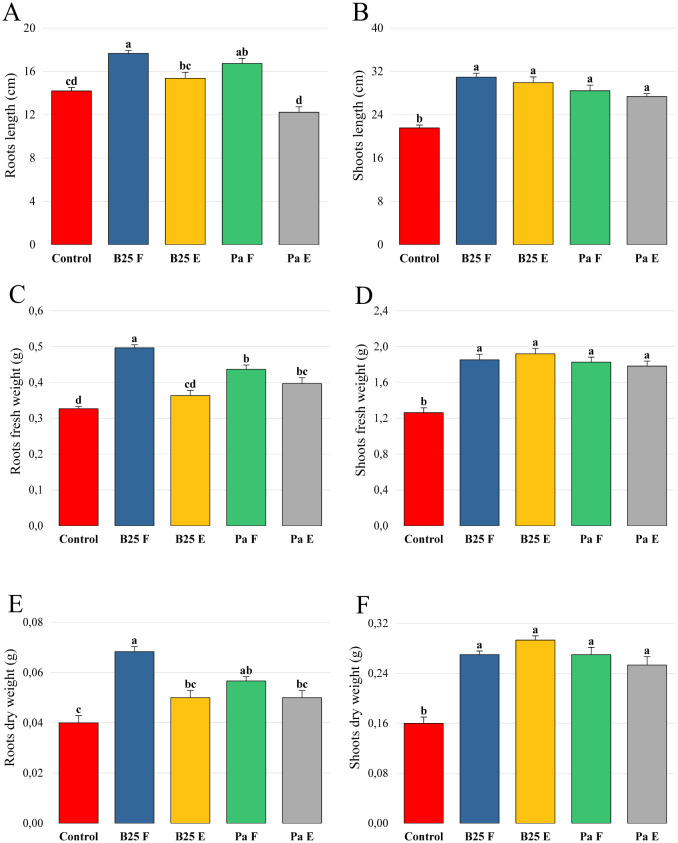
Effect of free and encapsulated bacterial inoculation on (A, B) roots and shoots length (cm), (C, D) roots and shoots fresh weight (g), and (E, F) roots and shoots dry weight of wheat plants. Bar plots represent the mean ± standard error of three experiments. OriginPro2022 was used to perform statistical analysis, using two-way ANOVA and Tukey's multiple comparison post-hoc test. The values corresponding to each treatment and not sharing the same letters are significantly different according to Tukey's HSD post-hoc test.

The evaluation of the application effect of the encapsulated bacterial strains on durum wheat plants was carried out using pot experiments under controlled conditions (Figure S3). The results of plant height and root and shoot fresh and dry weight are shown in Figure 9. Inoculation with the encapsulated strains significantly increased shoot height and fresh and dry weight compared to the negative control (Figure 9B,D,F). However, after examining the results of biomass and root elongation, this inoculation was found to have no significant effect (Figure 9A,C,E).

#### 3.6.2. Biochemical parameters

##### 3.6.2.1. Chlorophyll pigments

**Figure 10.**
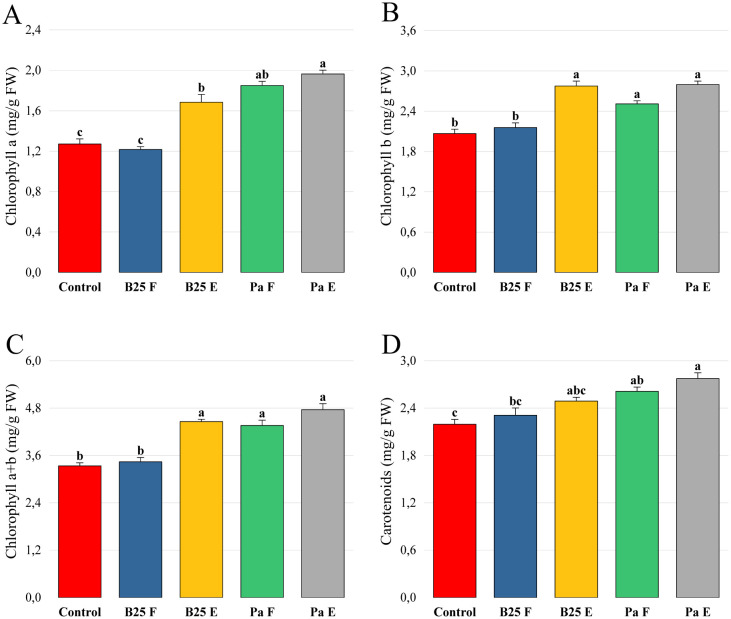
Effect of free and encapsulated bacterial inoculation on the content of (A) chlorophyll a (mg/g FW), (B) chlorophyll b (mg/g FW), (C) chlorophyll a+b (mg/g FW), and (D) carotenoids (mg/g FW) of wheat plants. Bar plots represent the mean ± standard error of three experiments. OriginPro2022 was used to perform statistical analysis, using two-way ANOVA and Tukey's multiple comparison post-hoc test. The values corresponding to each treatment and not sharing the same letters are significantly different according to Tukey's HSD post-hoc test.

Bacterial inoculation significantly improved the levels of chlorophyll pigments. The levels of chlorophyll a, b, total, and carotenoids were significantly lower in plants inoculated with the free strains than those inoculated with the encapsulated strains (Figure 10A–D). However, treatment with B25 in its free state had a non-significant effect compared to the negative control.

##### 3.6.2.2. Total sugars and proteins

**Figure 11.**
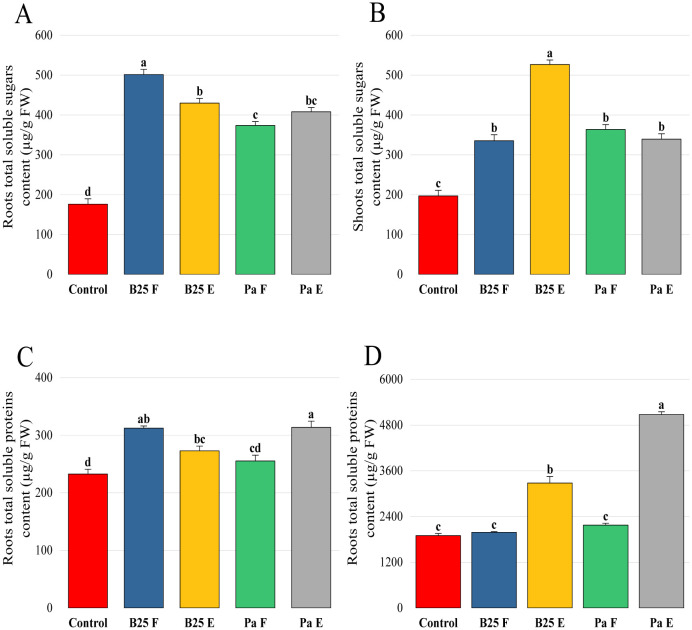
Effect of free and encapsulated bacterial inoculation on (A, B) the total soluble sugar content of roots and shoots (µg/g FW) and (C, D) the total protein content of roots and shoots (µg/g FW) of wheat plants. Bar plots represent the mean ± standard error of three experiments. OriginPro2022 was used to perform statistical analysis, using two-way ANOVA and Tukey's multiple comparison post-hoc test. The values corresponding to each treatment and not sharing the same letters are significantly different according to Tukey's HSD post-hoc test.

The total soluble sugar content of roots and shoots was significantly improved with inoculation compared to the control. This effect was not significant between the roots of plants treated with strains inoculated with free bacterial strains and those inoculated with encapsulated strains; on the other hand, the sugar content of shoots of plants inoculated with encapsulated B25 was significantly higher than those of any other treatment (Figure 11A,B). Concerning total proteins, our results indicate that the encapsulated strains strongly improved shoots' total proteins compared to free strains, while the free strain B25 and encapsulated Pa presented the best results regarding roots' total proteins (Figure 11C,D).

#### 3.6.3. Enumeration of rhizospheric, root epiphytic, and endophytic bacteria

**Figure 12.**
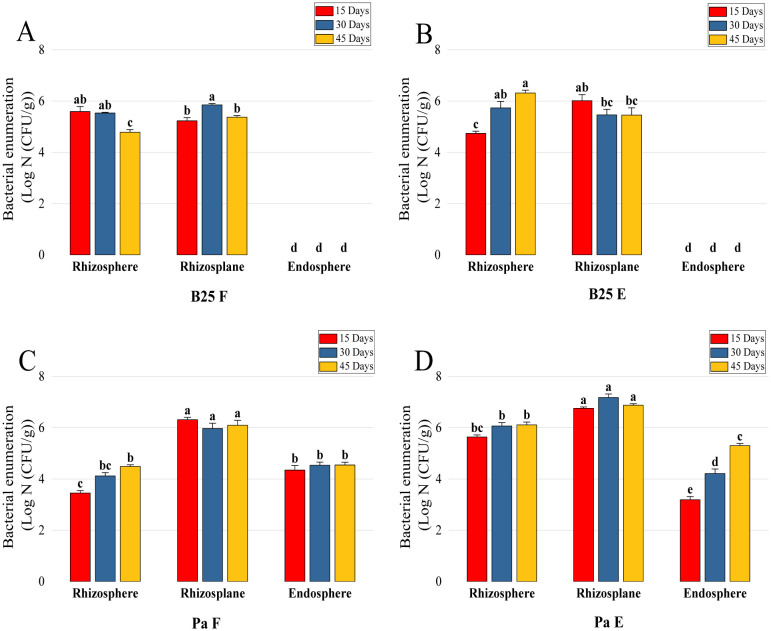
Bacterial enumerations (Log N (CFU/g)) of the rhizosphere, rhizoplane and endosphere of wheat plants inoculated with (A) free B25, (B) encapsulated B25, (C) free Pa, and (D) encapsulated Pa. Bar plots represent the mean ± standard error of three experiments. OriginPro2022 was used to perform statistical analysis, using two-way ANOVA and Tukey's multiple comparison post-hoc test. The values corresponding to each treatment and not sharing the same letters are significantly different according to Tukey's HSD post-hoc test.

Rhizospheric, root epiphytic, and endophytic bacterial density was analyzed after 15, 30, and 45 days of treatment. The results indicate a considerable decrease in the B25 load in the rhizosphere over time when freely inoculated, whereas it increased significantly after 45 days and reached 2.06 × 10^6^ (±0.18 × 10^6^) CFU/g in the case of encapsulated B25 (Figure 12A,B). Looking at rhizoplane-colonizing bacteria, the analysis revealed that there were no significant differences between the treatment of free and encapsulated B25; in both cases, endophytes could not be reisolated (Figure 12A,B). The bacterial survival of Pa in the rhizosphere was found to be lower when inoculated in the free form compared to the encapsulated form, which reached 3.1 × 10^4^ (±0.14 × 10^4^) CFU/g and 1.3 × 10^6^ (±0.02 × 10^6^) CFU/g, respectively (Figure 12C,D). The rhizoplane load of free and encapsulated Pa and the endophytes of free Pa were not modified as a function of time, while the endophytes of the encapsulated treatment increased notably, the enumeration of which was 2.01 × 10^5^ (±0.08 × 10^5^) CFU/g (Figure 12C,D).

#### 3.6.4. Evolution of weight and length of roots and shoots

**Figure 13.**
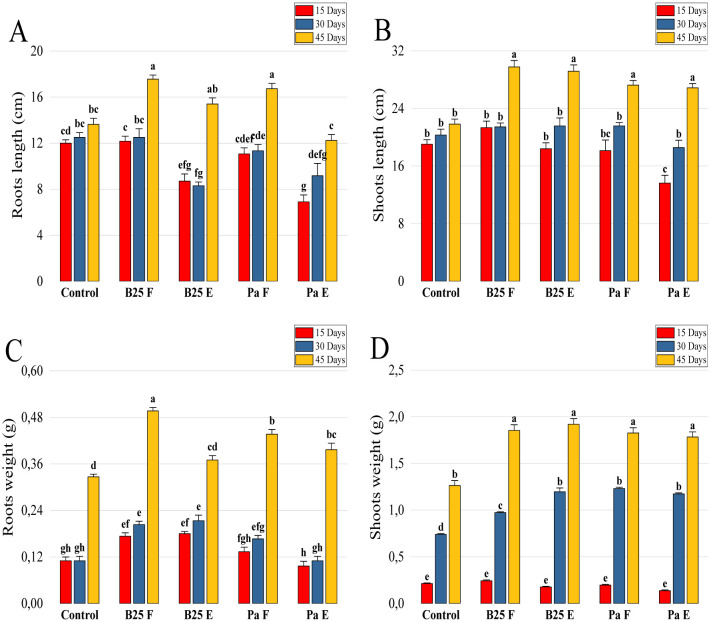
Effects of inoculation with free B25, encapsulated B25, free Pa, and encapsulated Pa on the length (A) of roots and (B) shoots, and the weight (C) of roots and (D) shoots of wheat plants after 15, 30, and 45 days of treatment. Bar plots represent the mean ± standard error of three experiments. OriginPro2022 was used to perform statistical analysis, using two-way ANOVA and Tukey's multiple comparison post-hoc test. The values corresponding to each treatment and not sharing the same letters are significantly different according to Tukey's HSD post-hoc test.

Length analysis of shoots and roots over time indicates a non-significant difference between the inoculated treatments and the control after 15 and 30 days (Figure 13). The effects of the treatments were more marked after 45 days, where both strains, whether free or encapsulated, significantly improved the weight and length of the roots compared to the control (Figure 13A,C). The encapsulated B25 treatment (B25 E) showed more pronounced effects on roots' length than the same treatment of Pa. For shoots, all treatments improved length and weight compared to the control, without a significant difference between free and encapsulated strains (Figure 13B,D).

## 4. Discussion

In order to develop an encapsulated bioinoculant, *Bacillus thuringiensis* strain B25 and *Pantoea agglomerans* strain Pa were encapsulated in a sodium alginate matrix. Encapsulation allows for an improved distribution of bacteria in the rhizosphere, thereby stimulating plant growth and health while reducing the harmful use of chemical fertilizers.

Since drying is the central step of the bioencapsulation process, a step that particularly impacts the viability of non-spore-forming bacteria [36], the addition of osmoprotectants could play a crucial role in improving cell survival during this process. Various osmoprotectants were tested in the presence of 50% PEG_6000,_ which was used here to create artificial drought stress similar to the drying stage of the encapsulation process, and proline, which significantly improved the growth of the two tested strains. Proline is known to be a very good osmolyte and a key osmoprotectant against water stress [37], and it has been shown to effectively maintain cellular integrity by interfering with membrane enzymes and proteins during water shortage [38]. Moreover, proline is an excellent stress-signaling molecule and antioxidant [38]. For these reasons, proline was added as a co-formulant in beads for the delivery of the two strains *B. thuringiensis* strain B25 and *P. agglomerans* strain Pa.

The introduction of the matrix solution containing sodium alginate, proline, and the B25 or Pa bacteria or their consortium in a CaCl_2_ solution successfully resulted in the formation of spherical and insoluble capsules, which confirms that alginate constitutes an effective matrix for trapping bacterial cells and proves that ionic gelation is a valid, safe, and successful method for the encapsulation of PGPR. A remarkable encapsulation efficiency was obtained (over 99%) for all treatments; these rates are significantly higher than those reported by Panichikkal et al. [27], Wu et al. [39], and Kaur et al. [40], which are 90%, 93%, and 94.11%, respectively. The combination of optimized encapsulation conditions, favorable characteristics of the bacterial strains, and precise handling techniques in our study seems to have been more optimized compared to previously mentioned studies and to have contributed to a higher efficiency of encapsulation. The optimal concentration of alginate can ensure a more homogeneous gel formation, capable of effectively retaining bacterial cells. The method of incorporation of the bacteria into the alginate and the solidification conditions, such as agitation speed during bead preparation and incubation duration in the presence of CaCl_2_, can also optimize the process and favor a better crosslinking of the alginate gel, thus reducing cell losses during encapsulation. Analysis of bacterial load in the dried beads showed that considerable survival rates were obtained after drying, higher than those of several previous studies. For instance, rates varying between 71.6% and 72.4% were obtained by Chi et al. [28]. This indicates that dehydration of the beads resulted in only a small cell loss. Berninger et al. [36] observed that bacterial survival during drying in the presence of osmoprotectants is much greater than in their absence; as such, we hypothesize that the higher survival reported by our study can be attributed to the presence of proline, which reduced cell loss and maintained water balance during the drying stage, particularly for the Gram-negative bacteria Pa.

The choice of drying protocol is also critical for the non-spore-forming survival, given the temperature and processing time. Deleterious lesions can occur, ranging from the formation of reactive oxygen species (ROS) to oxidative damage [41], depending on the technique used, knowing that the processing speed is proportional to the temperature [36]. Our results demonstrate that despite the considerable time of air drying (12 hours on average), our technique turns out to be profitable in terms of yield and cost, especially since it does not require any special equipment, unlike other techniques such as spray-drying, fluidized bed-drying, or vacuum-drying. Also, the addition of proline as an osmoprotectant provided a protective effect as an antioxidant and ROS inhibitor [38]. These findings are confirmed by the results of Berninger et al. [36], who reported that the addition of gum arabic and yeast extract as osmoprotectants significantly improved the viability of *Paraburkholderia phytofirmans* during drying.

In this work, bacterial cell survival in dry beads during storage at 4 °C was monitored. Our data showed that the number of viable cells of B25 remained constant at 2.76 × 10^7^ (±0.68 × 10^7^) CFU/g, while Pa showed a progressive decline in cell survival upon storage. On the other hand, the survival rate was lower for the two strains when encapsulated together rather than alone. These findings are in agreement with the literature since cell viability after encapsulation differs depending on the microorganism [42]. Storage temperature is another key factor that influences survival during storage [36]. Low temperatures have been shown to preserve the metabolism and energy of formulated bacteria, as evidenced by results obtained by Liffourrena and Lucchesi [43], where no significant loss of viability of the *Pseudomonas putida* bacteria was observed after 150 days of storage at 4 °C encapsulated in an alginate and perlite matrix. However, these findings cannot be generalized; in another study, the same *P.putida* bacteria encapsulated in 1.5% alginate and stored at 4 °C for 90 days presented a survival of only 0.3% [39], while a survival rate of 46% was observed in a bioformulation of *Bacillus megaterium* obtained by freeze drying stored at 4 °C for 6 months [28]. Other factors are involved in the stability during storage such as exposure to light, oxygenation, and packaging conditions, and their optimization should be taken into consideration [36]. It is important to point out that unlike in our study; few studies have reported the monitoring of viability over long periods even though this is necessary for the commercialization of bioinoculants [44]. Likewise, previous studies have reported encapsulation of mixed cultures [45],[46] but no investigation of long-term survival has been reported. The development of mixed bioinoculants should consider several issues, namely the absence of antagonism between the selected strains, similarity of physiological adaptation, adequate cellular concentrations, and compatibility with the genotype of the host plant [47]. Our study intended to contribute to this knowledge gap, developing encapsulated bacterial mixture formulations and investigating their long-term survival.

Another very important point addressed in this study regarding the application of biofertilizers in the field is demonstrating that the encapsulated bacteria can maintain their PGP activities during storage. The ability of Pa to produce IAA has been described previously [14]. Pa cells immobilized in alginate beads partially retain their IAA production capacities; this is in agreement with the observations of Ozdal et al. [48] and Panichikkal et al. [27]. There are no reports in the literature of monitoring this activity after storage of encapsulated PGP strains. The decline recorded is probably due to stressful storage conditions; the effect of lack of water on IAA synthesis has been previously discussed [49]. In addition, IAA is synthesized by very complex pathways and involves the intervention of several inducible enzymes, some of which can be repressed by environmental conditions [50].

The ability to produce siderophores was tested for both the bacterial strains B25 and Pa. They showed such ability during storage at variable rates with a decrease in production for B25. This could be likely explained by the inhibitory effect of osmotic stress on the gene responsible for bacillibactin synthesis [51], which are the predominant siderophores in *Bacillus* species [52].

*Bacillus* and *Pantoea*, the genera to which our strains belong, include the most efficient bacteria in phosphate solubilization [53]. The difference in phosphate solubilization recorded between B25 and Pa can be attributed to the physiological and molecular state of the two bacteria and to the diversity and efficiency of the solubilization mechanisms, which are intrinsically linked to the bacterial species [54]. The ability of Pa to maintain a bacterial concentration capable of producing secondary metabolites and to produce a diverse and efficient range of organic acids explains its superior performance in phosphate solubilization compared to B25. *Pantoea* species produce a variety of organic acids including citric, malic, succinic, acetic, oxalic, and formic acids [55]. These acids, which are mainly tricarboxylic and dicarboxylic, are generally more effective in solubilizing phosphates than mono and aromatic acids [56]. In comparison, a *Bacillus* strain isolated from wheat rhizosphere soil mainly produces glycolic acid, which is less effective for phosphate solubilization compared to other acids produced by *Pantoea* strains [57]. In addition, *Pantoea* also produces the phosphate hydrolysis enzyme phytase, adding another mechanism for the solubilization of phosphate that is not observed in *Bacillus* [58]. Also, Park et al. (2016) reported that no significant effect of co-inoculation compared to single inoculation was observed regarding phosphate solubilization [59].

The study of swelling properties revealed variable rates ranging from 209% up to 591%. These rates are similar to those obtained by Tu et al. (2016), which varied between 182% and 454% in an alginate and bentonite formulation [60]. The swelling kinetics is mediated by the water diffusion inside the bead and causes the release of alginate network chains [26]. Our observations indicate that the capsules rapidly absorb water, resulting in rapid swelling after the first day, and the interactions of the –OH groups of sodium alginate with water molecules progressively increase the rate of swelling of the beads due to the hydrophilic nature of alginate until saturation, which results in equilibrium after day 9 [61]. The swelling rates obtained in our study are relatively higher than those reported by He et al. [62] and Pour et al. [63]. Additionally, *Pantoea agglomerans* and *Bacillus thuringiensis* have been studied for their ability to produce exopolysaccharides (EPS) under standard culture conditions [64],[65]. These EPS, having hydrophilic properties, can amplify the beads' ability to absorb and retain water. According to Donati et al. (2005), the incorporation of hydrophilic substances into an alginate matrix can increase its swelling capacity due to increased water retention by EPS [66].

The release kinetics and cumulative survival of the encapsulated strains over a period of 60 days were carried out at room temperature. The obtained results are in agreement with those of Pour et al. (2019) [63]; the bacterial release is rapid for the first days and increases over time, the rapid diffusion of water inside the beads leads to expansion, and bacteria diffuse in the opposite direction of water dynamics [62]. This suggests that swelling contributes to this release by acting on the relaxation of the alginate matrix network by increasing pores size through which bacteria will pass from the interior of the bead to the exterior environment. The release slows down and then stabilizes due to the swelling balance and saturation of the capsules with water, which leads to a reduction of the channels at the level of the alginate envelope [67]. Encapsulation efficiency also influences release, according to Pour et al. [63] and Wu et al. [26], since a high cell density in the beads results in the release of a significant number of live bacteria.

The decrease in release rate can be attributed to environmental conditions and possible bacterial mortality [63]. The described ability of the capsules to effectively retain and release the bacteria object of this study suggests that the setup bioformulation could be successfully applied in the real scenario.

To the best of our knowledge, our study was the first to propose a model system to study the capacity to promote plant growth and the dynamics of root colonization of encapsulated bacteria in short-term experiments. Our investigations on *Arabidopsis thaliana* morphological parameters, root colonization, leaf area, and chlorophyll pigment dosage indicate that bacterial bioformulation in alginate beads provides a successful bioinoculant system.

It is important to point out that according to the literature, the exercise of PGP effects requires that bacteria come out of the bead and move toward the roots due to their ability to communicate with the plant via root exudates [68]. This communication is called chemotaxis and leads to root colonization [68]. Several factors are involved in the success of root colonization by favorable microbes, such as the biochemistry of the root surface and the biochemical composition of root exudates [69]. Thus, it was demonstrated during a study on the colonization of *Arabidopsis thaliana* roots by the rhizospheric bacterium *Pantoea* sp. YR343 that active metabolites such as auxins would be involved in the colonization process [70]. Similarly, some plant hormones can travel from the root to the leaves while enhancing the leaf growth process [68]. These data lead us to explain the improvement in weight, leaf surface, and chlorophyll pigment content in the leaves of inoculated *Arabidopsis thaliana* seedlings compared to those of the control, particularly in Pa whose profile of hormones and auxins such as cytokinins, indole acetic acid, and phenylacetic acid has already been described [14]. Nevertheless, the mobility of bacteria is necessary to adhere to the roots, and this mobility is induced by root exudates that serve as attraction molecules [68]. Parallel to this, a previous study has established that biofilm formation is essential for root colonization in *Bacillus* and that it is triggered by polysaccharides and other metabolites released by the plant [71]. Our data show that encapsulated bacteria significantly improve *Arabidopsis thaliana* growth compared to free inoculated strains, and this improvement is probably attributed to the increased efficiency of encapsulated bacteria in terms of colonization, mobility, and survival.

The rosette color of seedlings was darker under treatment with encapsulated bacterial strains compared to non-inoculated treatment. This is directly related to the process of photosynthesis, which is essential in plants to obtain energy, and its efficiency is positively correlated to chlorophyll content [72]. The darkening of the shoots of inoculated seedlings will therefore be explained by the increased action of encapsulated bacteria on the photosystem of *Arabidopsis thaliana*, whose chlorophyll pigments absorb light and convert it into chemical energy [73].

Before recommendation for field application, it is important to prove the plant probiotic functions of the developed formulations on a crop of high agriculture relevance. As such, we focused on durum wheat as the model crop. Our results confirm the previously studied biofertilizing capacity of the two tested strains [25]. A direct stimulation of plant growth was noted, in particular of the aerial system of the vegetative apparatus where a significant increase in length and weight of shoots was observed in plants treated with Pa and B25 in both free and encapsulated forms compared to control plants. These results validate the beneficial interactions between the tested bacteria and the host plant and describe that such capacity is maintained when the strains are delivered to plants as encapsulated cells in beads. Since shoot growth is the most important parameter for wheat yield, our study clearly demonstrates that the application of suspended or encapsulated strains similarly improves shoot growth; we can hypothesize that it would also improve wheat yield. In *Bacillus* species, this effect is directly linked not only to the production of phytohormones, siderophores, lipopeptides, polysaccharides, and enzymes [74] but also to the regulation of homeostasis and antioxidant enzymes in natural plant growth conditions and under various stresses [75]. In this sense, the capacity of B25 to fix nitrogen, produce NH_3_, ACC deaminase, and siderophores, and solubilize phosphate has already been discussed [25]. A similar study on the inoculation of encapsulated strains of *Bacillus* reported improved growth and development of durum wheat plants by increasing aboveground biomass and plant height; this benefit is mainly attributed to two key mechanisms: improved nutrient and mineral uptake (mainly N) from the soil, and the increased stimulation of the production of phytohormones involved in cell division and shoot elongation such as cytokinins and auxins [76]. Likewise, Pa proves effective in the production of siderophores, IAA, ACC deaminase, NH_3_ and HCN and in phosphate solubilization and nitrogen fixation [27]. In addition, *Pantoea* plays a stimulating role in the growth of cereal crops by increasing the availability and assimilation of nutrients while promoting more efficient regulation of endogenous phytohormones [77]. Moreover, it was found that even if the bacteria produces little or no phytohormones, its inoculation with wheat can induce and regulate phytohormone production by the inoculated plant [74]. Similarly, a previous study reported that the inoculation of a strain of *Bacillus* sp. V2026 altered endogenous IAA levels in shoot and root tissues and boosted wheat plant productivity and yield [74]. Another study revealed a strong correlation between the presence of cytokinin and shoot length, fresh weight, and dry weight, while bacterial IAA was negatively correlated with root length [78].

High concentrations of chlorophyll pigments indicate active metabolism and proper functioning of the plant's photosynthetic apparatus [74]. Inoculation of encapsulated bacteria improved the photosynthetic performance of durum wheat; this is probably associated with the effects of these bacteria in improving water and nutrient absorption, which play an important role in the structural construction of the photosynthetic machinery [79], and ensuring gas exchange and electron transport [80]. Thus, the production of IAA and ACC deaminase and the increase in plant biomass would also be involved in chlorophyll biosynthesis [81]. Indeed, the increase in carotenoid contents is linked to the collection of light energy necessary for photosynthesis [82].

Colonization capacity is an important trait that determines the ability of the bacteria to survive and compete with other microbes and defines the effectiveness and performance of PGPB during its inoculation in the field [83]. It is also considered the main survival factor of bacteria [83]. For this purpose, the colonization capacities of the rhizosphere, the rhizoplane, and the root internal tissues were analyzed. Our results demonstrate that both bacterial strains survived effectively in a sterile and relatively nutrient-poor medium and indicate an increase in the number of rhizospheric and endophytic bacteria over time in the treatments of encapsulated formulations, while a considerable decrease was observed in liquid formulations. These observations are consistent with those of Bhise and Dandge (2019), who found increased colonization of rice roots by a strain of *Pantoea agglomerans* encapsulated in alginate after 20 and 30 days compared to the same free inoculums [84]. In a different study focusing on cotton root colonization, the capacity of encapsulated *Pseudomonas putida* Rs-198 to colonize the roots significantly increased over time when compared with the inoculum of free cells [85]. Likewise, the analysis of the enumeration revealed that bacterial Pa loads are in agreement with the literature reports, being 10^6^ up to 10^9^ CFU/g in the rhizosphere and 10^4^ up to 10^8^ CFU/g in the root endosphere [86]. Considering that colonization is a complex process that involves the mobility of bacteria and their ability to chemotaxis with the host plant [87], the maintenance of these interactions is crucial to establish long-term stable bacterial associations with the host plant [86]. This ability to persist is strongly pronounced in encapsulated bacteria, which are slowly released into the soil, thus ensuring their survival and ability to promote plant growth, which guarantees their long-term yield. On the other hand, the capacity for rhizospheric and root colonization can be limited by the affinity to the genotype of the host plant and by competition with its internal microbiota when it comes to the endosphere of the plant [88]. The absence of B25 cells in the internal tissues of durum wheat suggests that the root endosphere does not belong to the privileged ecological zones of this strain. Furthermore, the analysis of morphological parameters over time suggests that the effect of inoculation on growth stimulation may be affected after more than 30 days of plant growth.

## 5. Conclusions

This work demonstrated the feasibility of developing a microbial biostimulant by the encapsulation of two bacterial strains, namely *Bacillus thuringiensis* strain B25 and *Pantoea agglomerans* strain Pa, in sodium alginate using the ionic gelation technique. Our results indicate that the addition of proline boosts bacterial survival during the drying process and storage and that alginate constitutes a sovereign system to trap and slowly release encapsulated bacteria.The bioformulations developed showed a notable survival rate over a 24-month storage period and demonstrated to effectively preserve plant probiotic activities played by the strains after being encapsulated in dry alginate beads for up to 24 months, both alone and in combination. The effectiveness of encapsulated bacteria in promoting the growth of *Arabidopsis thaliana* and durum wheat as well as their colonization capacity and kinetics were elucidated, moving forward in the direction of sustainable agriculture. Future research on field trials and natural conditions could further clarify the effectiveness of the setup delivery system under real scenarios.

## Use of AI tools declaration

The authors declare they have not used Artificial Intelligence (AI) tools in the creation of this article.


