# Supplementary material for: Root colonization dynamics of alginate encapsulated rhizobacteria: implications for Arabidopsis thaliana root growth and durum wheat performance
Source: AIMS Microbiol. 2025 Feb 5;11(1):87–125. doi: 10.3934/microbiol.2025006 (PMC11950683; doi:10.3934/microbiol.2025006)
Supplement: Supplementary file 1 [file microbiol-11-01-006-s001.pdf]

Microbiology

AIMS Microbiology, 11(1): 87–125.
DOI: 10.3934/microbiol.2025006
Received: 04 September 2024
Revised: 11 January 2025
Accepted: 20 January 2025
Published: 05 February 2025

https://www.aimspress.com/journal/microbiology

Research Article

Root colonization dynamics of alginate encapsulated rhizobacteria: implications for *Arabidopsis thaliana* root growth and durum wheat performance

Amel Balla[1], Allaoua Silini[1,*], Hafsa Cherif-Silini[1], Francesca Mapelli[2] and Sara Borin[2]

[1] Laboratory of Applied Microbiology, Department of Microbiology, Faculty of Natural and Life Sciences, University Ferhat Abbas of Setif -1, 19000 Setif, Algeria
[2] Department of Food, Environmental and Nutritional Sciences (DeFENS), University of Milan, 20133 Milan, Italy

* **Correspondence:** Email: siliniallaoua@univ-setif.dz; Tel: +213(0)775133491.

Supplementary

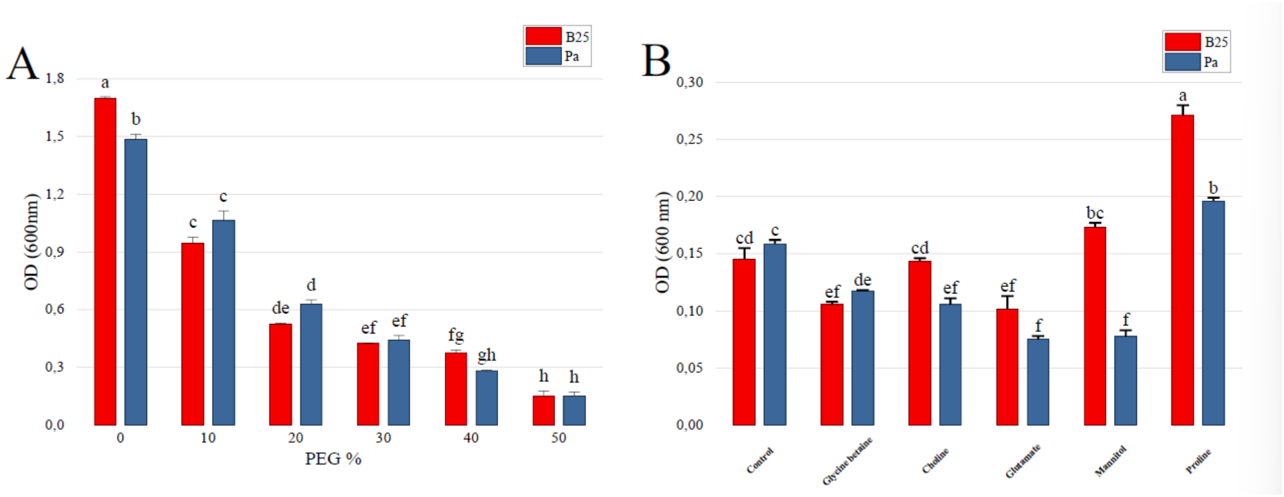

Figure S1. Effect of (A) different concentrations of PEG6000 (%) and (B) different osmoprotectants (mM) on the growth of strains B25 and Pa. Bar plots represent the average standard error of three different experiments. OriginPro was used to perform statistical analysis, using two-way ANOVA and Tukey's multiple comparison post-test. The effects of PEG6000 corresponding to each treatment and not sharing the same letters are significantly different according to Tukey's post hoc HSD test.

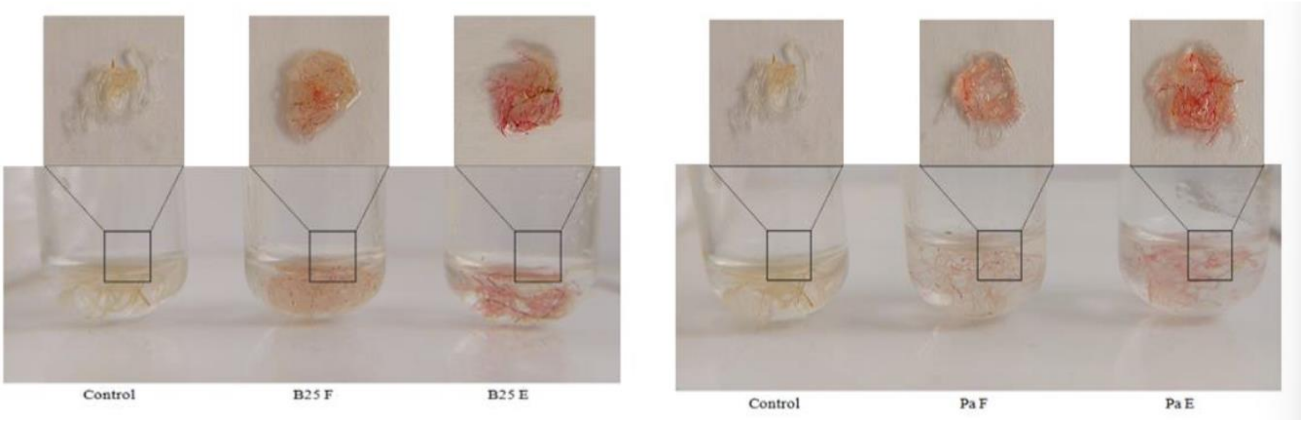

Figure S2. Illustration of *Arabidopsis thaliana* roots colonization by free and encapsulated bacteria revealed by triphenyltetrazolium chloride (TTC).

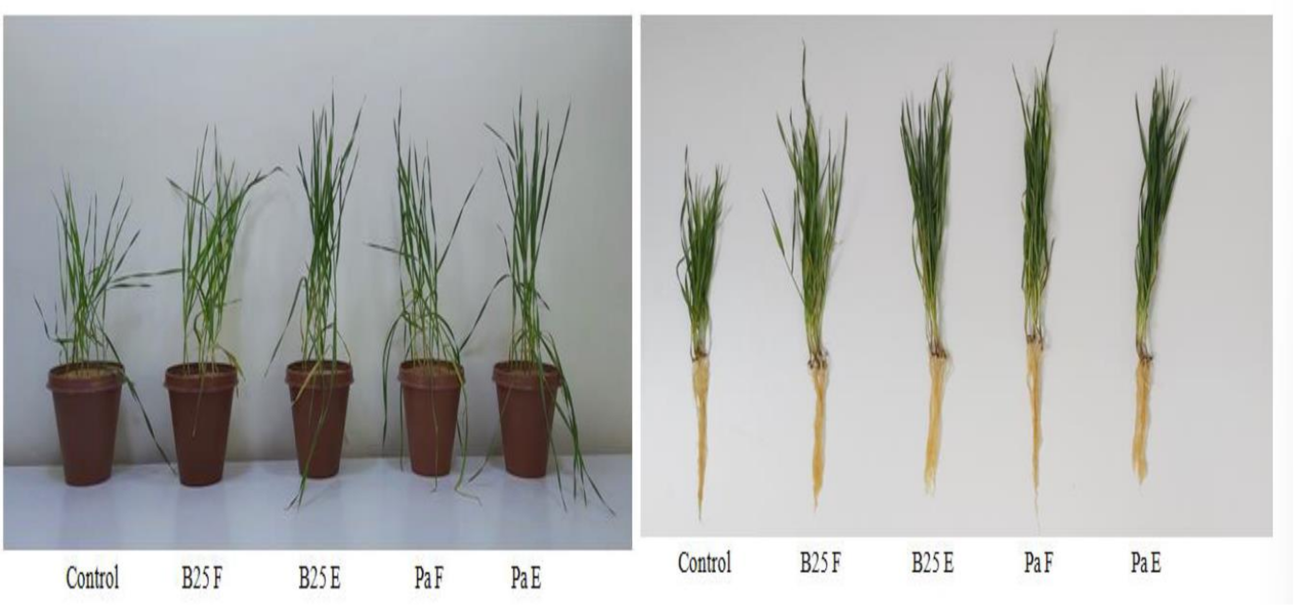

Figure S3. Inoculation effect of free and encapsulated B25 and Pa on durum wheat growth.

©2025 the Author(s), licensee AIMS Press. This is an open access article distributed under the terms of the Creative Commons Attribution License (https://creativecommons.org/licenses/by/4.0)
